# Promoting neuroplasticity and neuropsychological functioning in frailty through an app-based sensorimotor training: study protocol for a randomized trial

**DOI:** 10.1186/s12877-021-02293-9

**Published:** 2021-06-03

**Authors:** Florian Beier, Martin Löffler, Frauke Nees, Lucrezia Hausner, Lutz Frölich, Herta Flor

**Affiliations:** 1 Institute of Cognitive and Clinical Neuroscience, Central Institute of Mental Health, Medical Faculty Mannheim, Heidelberg University, Square J5, 68159 Mannheim, Germany; 2 Institute of Medical Psychology and Medical Sociology, University Medical Center Schleswig-Holstein, Kiel University, Kiel, Germany; 3 Department of Geriatric Psychiatry, Central Institute of Mental Health, Medical Faculty Mannheim, Heidelberg University, Mannheim, Germany; 4 Department of Psychology, School of Social Sciences, University of Mannheim, Mannheim, Germany

**Keywords:** Frailty, Neuroplasticity, Training app, Sensorimotor functioning, Aging

## Abstract

**Background:**

Frailty is characterized by an age-related decline in multiple physiological systems, leading to a high vulnerability to stressors, adverse health outcomes, and low quality of life. Neuroscientific models of pathological aging emphasize the loss of sensorimotor stimulation and reduced neuromodulatory capacities as core processes in age-related cognitive and bodily decline, which may be associated with maladaptive plastic changes in the brain. We plan to increase sensorimotor stimulation in frail persons through a newly developed app-based training program and link the training trials to biological and psychological correlates of age-associated vulnerability and health indices.

**Methods:**

We will conduct a randomized trial, applying an app-based sensorimotor home training (N = 30) in people suffering from frailty. An app-based relaxation training will serve as an active control condition (N = 30). Both interventions will last for 90 days each. The sensorimotor training includes unimodal and multimodal sensory discrimination tasks in the visual, auditory, and tactile domain, as well as sensorimotor precision tasks. The tasks will be implemented using an adaptive training algorithm and enriched with motivational components embedded in a virtual training environment. We expect a pre-post reduction of frailty status and associated functional decline related to refinement of representational maps within the sensorimotor system and improved sensorimotor function such as extremity function. Secondary analyses will study the influence of BDNF genotype as moderating variable. Additional outcomes will include measures of perceptual and cognitive functioning, quality of life as well as BDNF serum levels. Measurements will take place before training (baseline), after 60 days (assessment 1), and at the end of the training after 90 days (assessment 2).

**Discussion:**

In our randomized trial, we aim to characterize a multidimensional concept of frailty and to target maladaptive behaviors and neuroplasticity using an app-based sensorimotor training. This type of intervention might provide further knowledge and new possibilities for preventing decline and preserving function in older adults.

**Trial registration:**

ClinicalTrials.gov NCT03666039. Registered 11 September 2018 – Retrospectively registered.

Protocol version: Version 4 revised (issue date: 19 May 2021).

## Background

The term “frailty” refers to a clinical condition which is characterized by an increased vulnerability to stressors and poor health outcomes resulting from a cumulative decline in multiple physiological systems, such as the musculoskeletal, endocrine, and cardiovascular system [1, 2]. While a continuous decrease in physiological capacity also occurs with normal aging, this decrease is accelerated and exacerbated in frailty [3]. Neuroscientific models of pathological aging suggest that the brain might play a major role in determining healthy or pathological aging [4, 5]. For instance, age-related gray matter reductions in the frontal [6, 7] and medial-temporal [8, 9] regions have been associated with reduced gait and memory performance, while white matter atrophy in the corpus callosum is suggested to affect bihemispheric communication [10, 11]. From a functional perspective, aging is related to reduced neural differentiation and selectivity of perceptual [12, 13] and motor [14] representations. These altered representations in turn are thought to be involved in an age-related decline in cognitive performance, such as memory [15, 16] and sensorimotor performance, including upper extremity function, gait and balance [17, 18]. In this context, an important determinant is “disuse” of the brain, characterized by a reduction of perceptual inputs, motor actions, and cognitive stimulation that are required to refine existing skills and acquire new skills [5]. When people age they tend to stereotype and simplify behaviors and the brain is likely to adapt to these less complex behaviors by simplifying the underlying neuronal representations such as cortical sensorimotor maps [4, 5]. On the neuronal level, “disuse” of the brain is thought to lead to negative changes in neuronal metabolism, such as neurotransmitter production and function [19] and neuronal architecture, including the elaboration of dendrites, spines, and synapses [20]. Therefore, the fidelity and reliability of cortical representations is thought to decline, resulting in noisy neuronal processing in sensory and motor systems, which in turn might promote maladaptive behaviors such as motor instability, coordination deficits, movement slowing, inactivity, and social isolation. Low activity levels lead to an increase in peripheral and bodily symptoms, such as a loss of muscle mass, which would increase the risk of fall and fracture [21]. Together, these interrelated factors create a self-reinforcing downward spiral of altered brain function, physical disability and age-related functional decline.

Therefore, we assume that the brain might be at the center of the vicious cycle of frailty, starting with an initial impairment and leading to an accelerated decline of physical function. As the brain is a highly plastic organ that shows adaptive as well as maladaptive plasticity [22], it might be an optimal target for innovative interventions that utilize principles of neuroplasticity to delay or even reverse cortical and behavioral age-related changes.

To date, the majority of interventional studies in older frail individuals examine physical exercise protocols including aerobic [23] and muscular strengthening exercises [24]. Physical exercise has been suggested to improve physical performance such as muscle strength, balance, and gait speed [25], but also to enhance brain health and plasticity [26]. Physical therapy in pre-frail individuals was found to increase reduced plasma levels of brain-derived neurotrophic factor (BDNF), a neuronal growth factor involved in neurogenesis and synaptogenesis [27], which suggests a key role of neuromodulatory factors in mediating the syndrome of frailty [28]. However, only few of these studies have directly evaluated the influence of physical exercise on frailty itself [29] and the optimal procedure of how to prevent or reverse the syndrome of frailty is still a matter of debate [30, 31]. Moreover, despite the evidence suggesting a close link between structural as well as functional brain changes and physical decline during aging [17, 18], interventional studies targeting the relationship between brain structure and function and frailty are surprisingly rare. In healthy adults, neuroplasticity-oriented programs, including intensive sensory, cognitive, or motor stimulation, were shown to have the potential to strengthen neuromodulatory systems, promote beneficial neuroplasticity in cortical representations, and improve neurocognitive skills that decline with aging [32–34]. As motivational and affective processes seem to be less affected by maladaptive plasticity [35], these processes provide useful means to enhance training compliance and success. With respect to the multi-system decline observed in frail individuals, computerized neuroplasticity-oriented applications using virtual environments can be more useful because they can stimulate several systems at the same time, can give immediate feedback and permit the use of everyday activities including bodily activation that are relevant for the participant’s life [36]. Therefore, we describe a randomized trial in which we use an app-based multimodal sensorimotor training in frailty.

### Objectives of the study

Our hypothesis is that intensive forms of plasticity-oriented training can improve frailty. Our goal is to enhance relevant input to the sensory and motor brain systems in order to reverse structural and functional correlates of maladaptive neuroplasticity in cortical representational maps. We aim at counteracting the “disuse” of the brain, which should lead to positive changes in neuronal metabolism and architecture and thus to an increased fidelity and reliability of cortical representations and less noisy neuronal processing. In turn, we expect these neuroplastic changes to promote cognitive, physical, and sensorimotor function, leading to an improvement in frailty status and frailty-related health indices.

Based on previous plasticity-oriented training studies in healthy subjects [32–34], our training approach will consist of a multimodal training protocol including unimodal sensory discrimination and bimodal sensory integration tasks in the visual, auditory, and tactile domain as well as a sensorimotor precision tasks (cf. [37]). The training tasks will be implemented in an app-based manner and will be embedded in a motivating virtual environment including personally relevant reinforcers to counteract the age-related reduction in motivational drive and increase adherence to and efficacy of the training. To evaluate the specificity of the sensorimotor approach, we will implement a randomized controlled trial with an app-based relaxation training serving as an active control group.

We hypothesize that the sensorimotor training will have superior effects on frailty status compared with the control training, as reflected by a pre-to-post reduction in frailty indices and an improvement in frailty-related everyday functioning. On the neuronal level, we expect a refinement of sensorimotor representation maps and an increase in neuronal efficiency in the brain. Furthermore, we expect that such neuroplastic changes will be mirrored by a pre-to-post increase in BDNF serum levels. In addition, we will compare frailty-associated outcomes between BDNF genotypes that were shown to differ in terms of both BDNF secretion and the magnitude of training-related improvement [27, 38]. In the sensorimotor domain, we expect an increase in movement speed as well as an improvement in lower extremity motor function such as balance, and upper extremity motor function such as dexterity. With regard to sensory abilities, we expect an increase in visual, tactile, and auditory performance scores. Lastly, we expect the sensorimotor training approach to enhance psychological wellbeing, as reflected by an increased perceived health status and quality of life.

## Methods

### Design

This randomized controlled trial will compare a tablet-based sensorimotor training (experimental group) and a tablet-based relaxation training (control group) in subjects suffering from frailty. Both conditions are conceptualized as a 90-day home-based intervention. Assessments will take place (1) before (baseline), (2) after 60 days (assessment 1) and (3) at the end of training (90 days; assessment 2) at the Institute of Cognitive and Clinical Neuroscience, Central Institute of Mental Health, Mannheim.

### Study population and recruitment

**Fig. 1:**
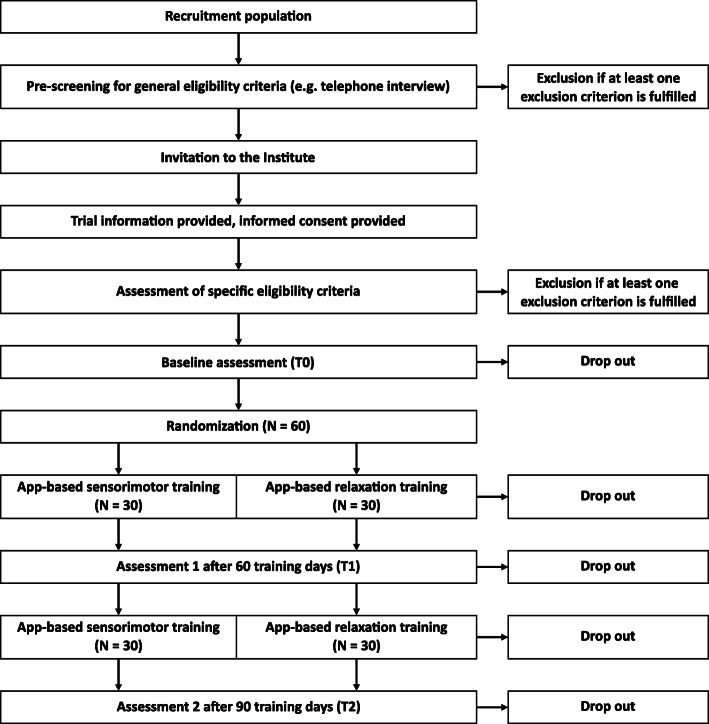
Expected flow of participants and study design

The expected recruitment of participants is shown in Fig. 1. Participants will be recruited from the general population as well as from geriatric hospitals via personal contact, newspaper advertisements, leaflets and online announcements. Prior to the invitation to the CIMH, potential subjects will be pre-screened for general eligibility via telephone interviews. Specific eligibility criteria will be tested and general eligibility criteria will be confirmed during the first examination appointment.

The inclusion criteria will be an age of 65 to 95 years and the presence of at least one of the five frailty phenotype criteria [39], i.e. self-reported unintentional weight loss of > 5 kg in the prior year, self-reported exhaustion, low level of physical activity specified in kcal/week, muscle weakness as measured by low grip strength using a Jamar hand dynamometer, and slowness by slowed gait speed over 4.57 m. According to the phenotype model, the presence of one or two criteria is considered as pre-frailty while the presence of three or more criteria is considered as frailty [39]. We will include pre-frail as well as frail persons to investigate neuroplasticity mechanisms across a broader range of age-related functional decline, as the pre-frail stage is considered to describe a condition at high risk of progression to frailty [40, 41] and may be more amenable to change. Exclusion criteria will be: acute bone fractures within the last 3 months, immobility, paralysis or confinement to bed; stroke or neurological disorders with major cognitive or physical impairments; dementia; myocardial infarction within the last 6 months; life-time prevalence of mental disorders such as schizophrenia and other psychotic disorders, bipolar disorder, obsessive-compulsive disorder, post-traumatic stress disorder, drug or alcohol addiction; current severe major depression or other acute axis 1 mental disorders; current intake of benzodiazepines or antipsychotics; vitamin B12-, folate- or thyroid-stimulating hormone deficiency. Specific exclusion criteria will be examined during the first visit and include: cognitive impairment defined as a Mini Mental State Examination (MMSE) score of ≤ 24; severe impairments in sensory abilities (i.e. visual acuity of < 0.1; mechanical detection threshold of > 512 mN; severe or profound hearing loss according to the WHO, defined as a mean hearing threshold of > 60 dB; severe tinnitus symptomatology).

### Procedure

Subjects fulfilling the general eligibility criteria as determined through the pre-screening will be invited for baseline assessments. During the first visit at the CIMH, subjects will provide written consent for participation. Specific eligibility criteria will be examined by a trained psychologist and a trained psychiatrist specialized in geriatric psychiatry. Individuals fulfilling the diagnostic criteria will then undergo behavioral, neuropsychological, and neurophysiological assessments conducted by the psychologist, and a physical and neuropsychological examination as well as the assessment of the medical history and medication conducted by the psychiatrist. Blood samples will be collected and conserved for analysis of BDNF serum levels and genotypes. After baseline assessments, subjects will be introduced to the respective training procedure by a psychologist. Support during the home-based treatment is also provided. After 60 and 90 days, the assessment of sensorimotor, neurocognitive and neurophysiological functioning will be repeated at the CIMH. Neuropsychological and physical examination including collection of blood samples will be repeated after 60 days. Prior to and after the training, participants will be asked for treatment expectation and evaluation, respectively, using five questions adapted from [42] to uncover motivational and affective aspects of the training. Subjects will not be paid for participation, but will be compensated for their travel costs.

### Randomization and blinding

Subjects will be randomly allocated on a 1:1 ratio to either the experimental or control condition. The randomization schedule will be generated electronically by a researcher not involved in the assessments. Group allocations will be kept in sequentially numbered sealed opaque envelopes. Participants will be informed about the random allocation procedure. Blinding of the investigator will not be possible because the investigator will conduct parts of the assessments and training procedures.

### Sample size

Previous studies in healthy older humans using sensory training have achieved promising results with medium effect sizes and sample sizes of 41 to 53 subjects [5, 32]. Given that our training includes a multimodal stimulation approach and focuses on motivational enhancement within a virtual training environment, we assume that in our participants the effect of the experimental training program would exceed a medium effect size. To estimate sample size, we use a repeated measures design with one between-factor with two groups and one within-factor with three assessment points [43]. Assuming a medium effect size of Cohen’s f = 0.25, 95% statistical power, a correlation of 0.50 between the dependent measurements, and a two-sided alpha error level of .05, we therefore would need 22 participants in each group. To account for a drop-out rate of about 25% during the training, we will include 30 subjects per group (60 in total).

### Interventions

#### Experimental condition

The interactive tablet-based sensorimotor intervention will consist of three consecutive training phases with each phase comprising 30 days (90 days in total). The first phase will consist of unimodal sensory discrimination tasks in the visual, auditory and tactile domain to increase distinctiveness and acuity of cortical sensory representations. In the second phase, subjects will be trained on bimodal sensory integration using visual-auditory, visual-tactile and auditory-tactile tasks. Bimodal sensory integration will require subjects to process stimuli of two different perceptual modalities and judge whether the two stimuli are synchronous or not. These tasks are assumed to increase temporal acuity for multisensory integration and to promote plasticity in perceptual integration brain networks [44, 45]. Unimodal and bimodal stimuli are presented through the tablet involving explicit answers on the tablet’s touch screen. The third phase will require participants to process bimodal sensory information in order to control a sensorimotor response, in the form of visual-auditory guided cycling, visual-tactile determined grasping, and auditory-visual controlled hands coordination. In these sensorimotor integration tasks, sensory input must be interpreted and integrated in terms of the current state of the motor system to continuously adjust motor commands and behavior [46]. These tasks aim at improving motor acuity, i.e. to increase precision and reduce variability of motor performance by increasing the signal-to-noise ratio in sensorimotor brain networks [47, 48]. For tactile, cycling, and grasping tasks, we will use external devices that are wirelessly connected to and controlled by the tablet (i.e. a Braille display device for tactile tasks, a customized ergometer for cycling tasks, and a handgrip dynamometer for grasping tasks).

Each daily training session will last 30 min allowing self-determined breaks in between. The training tasks will be embedded in a container application featuring a virtual environment of a gaming nature [34]. Performing the daily training tasks, participants can earn tickets to progress on a virtual journey throughout European cities. To enhance training motivation and efficacy, the program will include a customized application environment as well as personally relevant reinforcers (pictures, sounds) individually embedded in the tasks. To enhance training motivation, task difficulty will be dynamically adapted to provide a positive feedback percentage of 70–80%. Accordingly, the adaptive difficulty manipulation should produce sustained sensory and sensorimotor challenges, thereby promoting lasting neural changes and transfer effects [34, 49]. For monitoring and analysis purposes, training data including duration and achievements will be automatically collected by the app and sent to a dedicated server on a daily basis.

#### Control condition

The control intervention will be a self-developed tablet-based relaxation training encompassing daily 30-min sessions across a total training duration of 90 days, which do not include any of the critical features of the sensorimotor training app (i.e. multimodal sensorimotor training, adaptive algorithm, personalized feedback) using stimulating and variable exercises. Training sessions will require participants either to watch relaxation videos consisting of nature scenes or to follow verbally guided relaxation exercises, alternating on a regular basis. Relaxation exercises encompass various relaxation techniques, such as autogenic training, breathing meditation, mindfulness practice, or imaginary journeys. At the end of each session, participants complete a short questionnaire about their affective state including items from the German version of the Positive and Negative Affect Schedule (PANAS) [50, 51]. Training data collected by the app include responses to the post-training questionnaire as well as total amount of time engaged in the daily training.

### Outcome measures and biological moderators

The assessments will be performed according to the structure depicted in Table 1.

**Table 1:** Overview of outcomes, outcome measures, instruments and assessment time points

Outcomes	Outcome measures	Instruments	Assessment time points
**Personal information**	• Age, sex, education, clinical history, medication	• Self reports	• T0, T1, T2
**Frailty assessments**	• Frailty status (pre-frail, frail) • Frailty Index	• Frailty Phenotype • Frailty Index	• T0, T1, T2 • T0, T1, T2
**Brain plasticity in the sensorimotor and somatosensory system**	• Structural and functional parameters of cortical sensorimotor and somatosensory maps • MEP peak-to-peak amplitude and latency	• fMRI motor sequence task, fMRI somato-sensory mapping task • TMS at left primary motor cortex	• T0, T1 • T0, T1
**Biological markers of neuroplasticity**	• BDNF serum blood levels (ng/ml)	• Collection of blood samples	• T0, T1
**Sensorimotor performance**	• SPPB (total score) • CTSIB (total score) • PPT (means)	• SPPB • CTSIB • PPT	• T0, T1, T2 • T0, T1 • T0, T1, T2
**Sensory functioning**	• Visual acuity • Visual contrast sensitivity • Tactile grating thresholds • Fine-touch thresholds • Hearing thresholds	• FrACT • FrACT • JVP domes • Von-Frey filaments • Audiometer	• T0, T1, T2 • T0, T1, T2 • T0, T1, T2 • T0, T1 • T0, T1
**Cognitive testing**	• MMSE (total score) • Mental and motor response speed • Top-down attentional control • Visuospatial working memory capacity • Executive functioning	• MMSE • RTI • AST • SSP • IED	• T0, T1, T2 • T0, T1 • T0, T1, T2 • T0, T1 • T0, T1
**Functional level and quality of life**	• Functional level in frailty • Depression • Overall health status and quality of life • Functional capacity in everyday activities • Falls self-efficacy • Nutritional status	• FEFA • CES-D • SF-36, EQ-5D-5L • MKS • FES-I • MNA	• T0, T1, T2 • T0, T1 • T0, T1, T2 • T0, T1 • T0, T1, T2 • T0, T1

*AST* Attention Switching Task, *BDNF* Brain-derived neurotrophic factor, *CES-D* Center for Epidemiologic Studies Depression Scale, *CTSIB* Clinical Test of Sensory Integration of Balance, *EQ-5D-5L* EuroQol-5D-5L, *FEFA* Frail Elderly Functional Assessment, *FES-I* Falls Efficacy Scale – International Version, *fMRI* Functional magnetic resonance imaging, *FrACT* Freiburg Vision Test, *IED* Intra-Extra Dimensional Set Shift, *MEP* Motor-evoked potential, *MKS* Marburg Competency Scale (Marburger Kompetenz Skala), *MMSE* Mini Mental State Examination, *MNA* Mini Nutritional Assessment, *PPT* Purdue Pegboard Test, *RTI* Reaction Time, *SF-36* Short Form-36 Health Survey, *SPPB* Short Physical Performance Battery, *SSP* Spatial Span, *T0* Baseline assessment, *T1* Assessment 1 after 60 days, *T2* Assessment 2 after 90 days, *TMS* Transcranial magnetic stimulation

#### Frailty status

Comparison with other training studies will be possible by using the frailty phenotype [39] as a measure of frailty status as primary outcome. Additionally, we will assess the frailty index [52, 53], which is calculated as the ratio of the number of deficits present out of a total number of 40 deficits assessed, including previous diseases, disability, psychosocial risk factors as well as physical and cognitive impairments [53]. Thus, the frailty index consists of a continuous score between 0 and 1 with higher values representing more pronounced frailty and increased susceptibility to adverse health outcomes [53]. While some studies demonstrated that frailty scores obtained from the two measures are comparable [54, 55], others have suggested that the frailty index might discriminate better at the lower to middle end of the frailty continuum [56] and might be more sensitive to measure change after an intervention [57]. Therefore, we will use both measures in a complementary rather than comparative manner [58].

#### Brain plasticity in the sensorimotor and somatosensory system

In order to track correlates of functional and maladaptive neural plasticity, we will measure neural processing and cortical representations in the sensorimotor as well as somatosensory system using functional magnetic resonance imaging (MRI).

Sensorimotor brain activation will be investigated using a motor sequence task encompassing three different motor exercises of varying complexity [59]. During each trial, participants will have to perform a sequence of button presses with their right hand using an MRI-compatible keyboard with five keys that are numbered from 1 to 5 and correspond to the thumb, index finger, middle finger, ring finger, and little finger, respectively. The task consists of three different exercises requiring repetitive tapping of a certain sequence: the “FINGER” condition consists of repetitive tapping of key no. 2 with the index finger, the “SIMPLE” condition consists of the sequence 1–2–3-4-5, and the “COMPLEX” condition consists of the sequence 1–3–5-2-4. The task will be carried out in a block-wise manner consisting of alternating movement and rest blocks.

Functional MR scans will be recorded using a 3 T Magnetom Trio system (Siemens, Erlangen, Germany) and a 32-channel head coil. Using an EPI gradient echo sequence, we will collect 40 slices with 2.3 mm slice thickness, TE = 22 ms, TR = 2100 ms, FoV = 220 × 220 mm^2^, voxel resolution = 2.3 × 2.3 × 2.3 mm^3^.

To investigate neuronal reorganization in the somatosensory system, we will perform a spatial mapping of the finger representations in the primary somatosensory cortex (S1) using two different protocols of somatosensory stimulation. Stimuli will consist of pneumatic sensations of touch automatically applied at a frequency of 1 Hz through a custom-made pneumatic device [60]. In a first run, tactile stimuli to the five finger pads will be separately applied in a random block design interspersed with rest blocks. In a second run, we will apply a well validated phase-encoding paradigm in which fingers will be continuously stimulated with no rest blocks. This approach has been demonstrated to reveal highly reproducible maps of individual digits in S1 [61]. Stimulation will cycle through blocks of fingers in an ascending and descending order. For both somatosensory paradigms, we will use an EPI gradient echo sequence and collect 22 slices with 1.8 mm slice thickness, TE = 22 ms, TR = 1500 ms, FoV = 220 × 220 mm^2^, voxel resolution = 1.2 × 1.2 × 1.8 mm^3^. In addition, we will acquire T1-weighted structural scans (TE = 2.72 ms, TR = 1900 ms, FoV = 250 × 250 mm^2^, voxel resolution = 0.8 × 0.8 × 0.8 mm^3^) to perform voxel-based morphometry.

#### Neuronal efficiency in the sensorimotor brain system

Age-related decline in sensorimotor performance has been linked to decreased neuronal efficiency in the sensorimotor system with respect to reduced motor cortex excitability [62, 63]. To investigate motor cortex excitability at baseline and after the intervention, we will perform single-pulse transcranial magnetic stimulation (TMS) at the left primary motor cortex (M1) and simultaneous recording of motor-evoked potentials (MEP) at the right abductor pollicis brevis. As measures of motor cortex excitability, we will calculate MEP peak-to-peak amplitudes and latencies. Prior to the stimulation, we will determine individual resting motor threshold (RMT), defined as the stimulation intensity at which at least 6 out of 10 MEPs reached a peak-to-peak amplitude of ≥50 μV [64]. We will then conduct three stimulation runs at 100, 110 and 120% of the RMT intensity in pseudorandomized order. Each run will consist of 20 consecutive stimulations with an inter-stimulus interval of approximately 10 s.

#### Sensorimotor performance

To measure lower extremity function we will use the Short Physical Performance Battery (SPPB) [65] comprising measures of balance, walking speed, and sit-to-stand ability. Individual subtest scores range from 0 to 4, resulting in a summary score ranging from 0 to 12. Lower SPPB scores represent reduced physical abilities and have been associated with greater frailty [66] though previous studies demonstrated that physical activity interventions can result in improved SPPB scores [67].

We will use a modified version of the Clinical Test of Sensory Integration of Balance (CTSIB) [68] to estimate how well subjects can utilize vision, somatosensation, and vestibular information for the maintenance of postural stability. Subjects are required to maintain their feet side-by-side for 30 s with eyes open on a firm surface, eyes closed on a firm surface, eyes open on an unstable surface, and eyes closed on an unstable surface.

Upper extremity function will be assessed using the Purdue Pegboard Test (PPT) [69] requiring participants to place cylindrical metal pegs into holes either with the dominant, non-dominant or both hands simultaneously within a given time. In a fourth condition, participants have to combine pegs, washers and small tubes into a pre-defined assembly. Thus, the test measures fine and gross motor dexterity as well as coordination of hands, fingers, and arms [70] and poor PPT performance has been shown to be associated with age-related frailty [71].

#### Sensory functioning

In the sensory domain, we will assess visual, tactile, and auditory functioning. Visual testing will be carried out using the automated Freiburg Vision Test (FrACT) [72, 73]. For visual acuity testing, we will use Landolt Cs and Snellen Es of different size and orientation depicted on a computer screen in a pre-defined distance and under standardized lighting conditions. Visual contrast sensitivity will be assessed using Landolt Cs and gratings of different orientation and level of contrast.

Tactile discrimination performance will be tested in the form of a grating orientation task using hemispherical plastic domes (JVP Domes, Stoelting Europe, Dublin, Ireland), which have parallel bars and grooves of equal width on their surface (15 domes with a width range of 0.35 to 12 mm). To determine grating orientation thresholds, gratings are manually applied by the experimenter to the subjects’ skin (right index finger pad, right ring finger pad, right lower lip, in a randomized order) and subjects are to indicate the orientation of the grating (vertical, horizontal).

Fine-touch thresholds will be evaluated by probing the fingertips of the dominant second and fourth digit as well as the back of the dominant hand using von Frey filaments (Marstocknervtest, Marburg, Germany). Touch forces range from 0.25 mN to 512 mN on a logarithmic scale. Thresholds will be determined by using a staircase procedure [74].

To assess auditory acuity, hearing thresholds within the frequency range from 0.125 to 8 kHz will be determined in the form of an audiogram using a screening audiometer (MA 25, MAICO Diagnostics GmbH, Berlin, Germany). Based on a staircase procedure, single tones will be presented via headphones separately to the right and left ear in a counterbalanced sequence.

#### Cognitive testing

Previous research demonstrated that physical frailty might be associated with changes and impairments in a number of cognitive domains, such as episodic, semantic, and working memory [75], perceptual and psychomotor speed [75, 76], executive function [77] and top-down attention [78]. To assess cognitive abilities we will select four subtests of the *Cambridge Neuropsychological Test Automated Battery* (CANTAB; Cambridge Cognition (2019), www.cantab.com).

The *Reaction Time (RTI)* test assesses information processing speed, allowing for separate estimates of mental and motor response speed.

Using the *Attention Switching Task (AST)* we will examine top-down attentional control defined as the ability to flexibly switch attentional resources towards relevant information and inhibit irrelevant information. Dependent measures include response time and accuracy, switching cost and congruency cost.

Using the *Spatial Span (SSP)* test, we will assess working memory span to obtain an estimate of visuospatial working memory capacity.

Finally, we will investigate the integrity of fronto-striatal pathways using the *Intra-Extra Dimensional Set Shift (IED)*, which is a computerized version of the Wisconsin Card Sorting test. The IED is a test of executive functioning including rule acquisition and reversal as well as attentional set formation maintenance, shifting and flexibility of attention.

#### Functional level and quality of life

To assess function in frail elderly at a very low activity level, we will apply the Frail Elderly Functional Assessment (FEFA) [79], which has been demonstrated to be valid, reliable, and sensitive to change [80]. Using the German version of the Center for Epidemiologic Studies Depression Scale (CES-D), we will assess depressive symptoms experienced in the past week based on a 20-item self-report scale [81, 82]. Overall health status and quality of life will be evaluated using the Short Form-36 Health Survey (SF-36) [83] and the EuroQol-5D-5L (EQ-5D-5L) [84]. The SF-36 is a widely used survey of quality of life in health economics and allows for evaluation of patient health on physical and mental health scales. The EQ-5D-5L quantifies health status in five dimensions and on a visual analogue scale ranging from 0 to 100. To estimate functional capacity of the participants, we will use the self-report version of the Marburg Competency Scale (Marburger Kompetenz Skala, MKS) [85], which involves 30 questions on competence in typical everyday activities. Falls self-efficacy will be measured using the Falls Efficacy Scale – International Version (FES-I) [86], a self-report questionnaire assessing the level of concern regarding the possibility of falling when performing certain activities of daily living. To screen for malnutrition, we will use the Mini Nutritional Assessment (MNA) [87].

#### BDNF serum level assessment and genotyping

Blood samples collected by the study psychiatrist will be processed according to standard protocols and stored at − 80 °C until analysis. BDNF serum levels as a marker of synaptic plasticity will be measured using a highly sensitive fluorometric enzyme-linked immunoabsorbent assay (ELISA). For genetic analyses, we will extract DNA from blood samples. GWAS analysis will be performed on an Illumina GSA1.0 SharedCustom Content bead array according to the manufacturer’s instructions. GenomeStudio 2.0 software will be used to determine BDNF genotypes and results will be exported in PLINK format.

### Data analysis and handling

We will use mixed repeated measures analyses of variance for each of the outcome variables in order to assess the effect of the training at baseline, after 60 days, and after 90 days, as well as to unveil differences between the two groups. Significant time-by-group interactions will be further examined using post-hoc tests. To evaluate changes through the interventions, pre-post effect sizes will be computed and compared across interventions. The problem of missing data will be handled using multiple imputation.

Data collected in this project will be recorded, stored and analyzed anonymously. A list assigning codes to names will be stored separately under high security standards. Analysis of the data will be of scientific purpose only.

### Ethics and dissemination

The study has been approved by the local Ethics Committee (Medical Ethics Committee II, Medical Faculty Mannheim, Heidelberg University; 2015-544 N-MA) according to the Declaration of Helsinki and has been registered under the trial number NCT03666039. If any important changes apply to the trial protocol, the trial registration will be updated. Any personal information of study participants will be stored and protected in accordance with the most recent General Data Protection Regulation of the European Union and will be monitored by the department’s data protection commissioner. Direct access to data will be restricted to authorized representatives from the host institution and the regulatory authorities. The results of the study will be submitted for publication in a selected peer-reviewed journal on geriatrics and presented at relevant international conferences.

Participants will be informed about all procedures and written informed consent will be obtained. Any severe adverse events caused by our assessments or treatment protocols will be recorded and analyzed. During assessments, participants will be monitored by the investigator at all times and on a regular basis during the training procedures to maintain the opportunity to intervene if any unintended effects occur. A physician is available in cases of emergency. During measurements, the institute’s liability insurance protects the participants against mishandling of the investigator.

## Discussion

With the number of older people strongly increasing in almost all countries, frailty prevalence is expected to rise dramatically [2], placing a heavy burden on health and aged care systems [88, 89]. As clinical studies in frail older patients are challenging and still rare, or yielded inconsistent results, procedures to reliably identify early onset stages of frailty as well as appropriate interventions to prevent disability and preserve physical functions are desperately needed.

The objective of our intervention is to increase relevant input into the sensorimotor system in order to engage competitive processes in the brain that refine the representations of sensory inputs and motor actions, thereby increasing the strength of cortical resources and reversing the maladaptive “disuse” of the brain [5]. Therefore, we expect our approach to promote sensorimotor and physical abilities, including motor stability, gait, and extremity function, and to reduce maladaptive behaviors, such as inactivity and social isolation, thereby leading to an improvement in frailty status.

Previous interventional studies in frailty included various types of intervention, such as physical exercise [24, 90, 91], nutritional intervention [92, 93], cognitive training [94], or geriatric assessment intervention [95, 96]. In particular physical exercise interventions demonstrated to be effective in reducing frailty and improving physical performance, while other interventions revealed only small or no training effects [25, 29]. This heterogeneity of study results might be due to differences in the study protocols with respect to training protocol, intensity, frequency, delivering method, and outcome measures. Thus, the reasons why some interventions are effective while others are not still remain unclear. This impedes interpretability and generalizability of the findings [29].

Despite the fact that frailty is associated with a decline in multiple physiological systems [1], the role of the central nervous system and in particular the brain in the pathogenesis of frailty has been addressed in only a small number of studies and still remains unclear. In fact, evidence suggested a link between frailty and brain physiology. For instance, the physical frailty phenotype has been related to reduced cerebellar gray matter volume [97], and some of the phenotype criteria, particularly walking speed, have been associated to regional brain volumes in the prefrontal cortex [6] and corpus callosum [98]. Moreover, evidence suggested a clear link between frailty and cognition, such that the presence of frailty can predict cognitive impairment and neurodegenerative diseases, such as dementia, within a few years [99, 100]. In this context, neurotrophic factors, including BDNF, may play an important role in survival, due to their role in promoting the differentiation of new neurons and synapses [101] and in preventing neuronal death during stress [102]. In fact, physical therapy intervention was found to increase plasma BDNF levels in pre-frail elderly women, suggesting that BDNF may be a key mediator in the pathophysiology of frailty [28]. However, interventional studies in frailty focusing on training effects in the brain are still lacking.

With the current trial, we will employ a multidimensional characterization of frailty, including neurophysiological, physical, sensorimotor, cognitive, and functional variables, which will enable us to investigate training effects on frailty status as well as on frailty-related outcomes. Through our intervention, we will target core central nervous processing in the sensorimotor system, by which we intend to create a link between structural, functional and biological markers of neuroplasticity and potential changes in frailty status. This approach aims at identifying the underlying pathogenic mechanisms of frailty and the results may add to previous interventional studies by offering new possible explanations for training effects found in frailty status and peripheral physical outcomes, such as muscle strength and muscle mass. Moreover, our study will provide new insights into how to prevent the continuous decline and avoid adverse outcomes in the development of frailty. This is important because frailty is a condition, which evolves from a subclinical state into a state of decompensation and ultimately overt frailty in the presence of stressors [103, 104]. Depending on one’s preceding frailty state, transitions between frailty states may occur frequently over time and may also be recurrent, suggesting that frailty is a dynamic process [41]. By including frail as well as pre-frail subjects, we will be able to relate the training effects to changes in frailty status across a broad range of severity levels of pathological aging. In this context, our study might contribute to identify useful strategies, such as the maintenance of sensorimotor stimulation and function, for slowing progression in an early state and preserving older people’s physical functions, autonomy, and quality of life that future studies can be based on.

### Strengths and limitations

The present study has several strengths. First, the study includes an innovative multimodal sensorimotor approach designed to specifically target mechanisms of pathological neuroplasticity in frailty, compared to an active control condition. Second, participants will be recruited from the general population instead of clinical institutions. This will increase generalizability of the results and approach. Third, the treatment procedures are conceptualized as home-based training, thereby presumably increasing compliance for daily care.

The study is also subject to some limitations. Researchers and participants will not be blinded to treatment condition. However, by randomly assigning subjects to the treatment conditions and by using an appropriate active control group, we aim to eliminate any effects of expectations or systematic biases from inducing performance differences. The study design does not include follow-up assessments going beyond post-training measurements after completion of the training, which should be addressed in future studies.

## Data Availability

The datasets produced by the completed study will be available from the corresponding author on reasonable request.

## Abbreviations

AST: Attention Switching Task
BDNF: Brain-derived neurotrophic factor
CANTAB: Cambridge Neuropsychological Test Automated Battery
CES-D: Center for Epidemiologic Studies Depression Scale
CTSIB: Clinical Test of Sensory Integration of Balance
EQ-5D-5L: EuroQol-5D-5L
EPI: Echo-planar imaging
FEFA: Frail Elderly Functional Assessment
FES-I: Falls Efficacy Scale – International Version
FoV: Field of view
IED: Intra-Extra Dimensional Set Shift
M1: Primary motor cortex
MEP: Motor-evoked potential
MKS: Marburg Competency Scale (Marburger Kompetenz Skala)
MMSE: Mini Mental State Examination
MNA: Mini Nutritional Assessment
MRI: Magnetic resonance imaging
PANAS: Positive and Negative Affect Schedule
PPT: Purdue Pegboard Test
RMT: Resting motor threshold
RTI: Reaction Time
S1: Primary somatosensory cortex
SF-36: Short Form-36
SPPB: Short Physical Performance Battery
SSP: Spatial Span
T0: Baseline assessment
T1: Assessment 1
T2: Assessment 2
TE: Echo time
TMS: Transcranial magnetic stimulation
TR: Repetition time
